# Global burden and trend of acute lymphoblastic leukemia from 1990 to 2017

**DOI:** 10.18632/aging.103982

**Published:** 2020-11-16

**Authors:** Ming Yi, Linghui Zhou, Anping Li, Suxia Luo, Kongming Wu

**Affiliations:** 1 Department of Oncology, Tongji Hospital of Tongji Medical College, Huazhong University of Science and Technology, Wuhan, China; 2 Bone Marrow Transplantation Center, The First Affiliated Hospital, School of Medicine, Zhejiang University, Hangzhou, China; 3 Department of Medical Oncology, The Affiliated Cancer Hospital of Zhengzhou University and Henan Cancer Hospital, Zhengzhou, China

**Keywords:** global burden of disease, acute lymphoblastic leukemia, cancer statistics, social-demographic index, cancer risk factor

## Abstract

Acute lymphoblastic leukemia (ALL) is a common malignant hematologic disease that is characterized by large numbers of dedifferentiated lymphoid cells. Statistical data of ALL's incidence and mortality are fundamental for policymakers to allocate resources optimally. In this study, we reported the incidence, death, and disability-adjusted life year (DALY) of ALL in the globe from 1990 to 2017. Our analysis showed that the incidence case of ALL increased by 30.81%, while the age-standardized incidence rate (ASIR) maintained stable. Subgroup analysis by social-demographic index (SDI) showed that ALL's ASIR was significantly decreased in high SDI countries, but were moderately increased in high-middle SDI countries. The change trends of age-standardized death rate and DALY rate were similar to ASIR trends. Subgroup analysis by age groups showed that children and the elderly were more likely to suffer ALL. Risk factor analysis demonstrated that smoking was the most significant contributor to ALL's death and DALY in the globe. Besides, the high body-mass index is playing an increasingly important role in ALL-caused mortality. Multiple methods to counteract potential risk factors should be adopted, such as controlling body-mass index in all regions and avoiding occupational exposure to carcinogens in low SDI countries.

## INTRODUCTION

Acute lymphoblastic leukemia (ALL) is a commonly diagnosed hematologic tumor. In the United States, approximately 5930 cases of ALL were newly diagnosed, and nearly 1500 patients died from ALL in 2019 [1]. It has been well-established that ALL's incidence rate is closely related to age and gender [2]. Previous reports showed that the incidence rate of ALL reached a peak in 1~4-year-old children, then dropped sharply, and had the lowest point in 25~45-year-old adults [1, 3]. Around 60% of ALL cases are diagnosed before the age of 20 [2]. Compared to females, males are more likely to develop ALL (the ratio of male/female = 1.23, in the United States). The molecular basis of ALL is complex, including genetic and environmental risk factors [4–7]. Benefiting from developments of intensified chemotherapy as well as immunotherapeutic strategies such as Rituximab and chimeric antigen receptor T-cell therapy, the clinical outcomes of ALL patients have been significantly improved [8–10]. At the present stage, the 5-year survival rate of children with ALL is over 90% [11]. However, the clinical outcomes of ALL vary in different age groups and countries. Older adult patients tend to have a poor prognosis, and basic treatment of ALL might not be available in low- or middle-income countries [2]. A comprehensive analysis of ALL's global burden and incidence trend helps assess the health cost and make corresponding policies.

Global burden disease study 2017 (GBD 2017) dataset provides the burden of 354 diseases in 195 countries and regions [12, 13]. The incidence rate, death rate, and disability-adjusted life year (DALY) rate could adequately reflect the disease burden and potential public health cost. In this study, we collected statistic data of ALL's incidence rate, death rate, as well as DALY rate and analyzed their change trends in different regions and countries. Socio-demographic Index (SDI) is developed by GBD researchers, which is a comprehensive indicator of social development degree. For some tumor types, the incidence and death rates significantly change along with social development degree [14, 15]. Therefore, we explored the relationship between the change trends of ALL' burden and SDI.

## RESULTS

### The incidence trends of ALL

**Figure 1:**
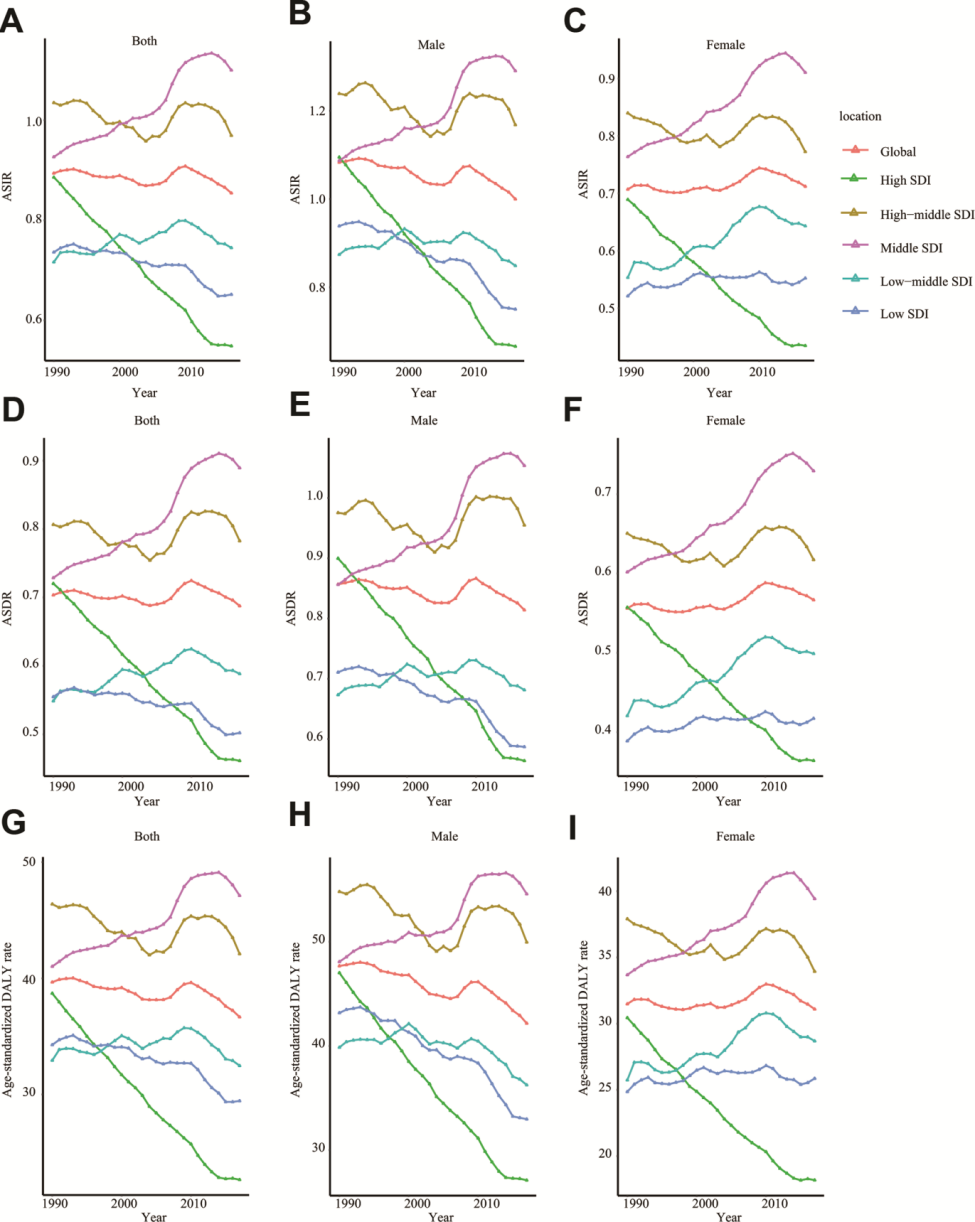
**The change trends of ASIR, ASDR, and age-standardized DALY rate.** ASIR of total population (**A**), male (**B**), and female (**C**); ASDR of total population (**D**), male (**E**), and female (**F**); age-standardized DALY rate of total population (**G**), male (**H**), and female (**I**). SDI: Socio-demographic Index; ASIR: age-standardized incidence rate; ASDR: age-standardized death rate; EAPC: estimated annual percentage change.

In the globe, the incidence cases of ALL increased from 49.07*10^3^ in 1990 to 64.19*10^3^ in 2017 (Table 1). However, the ASIR (per 100,000 individuals) kept stable (from 0.89 in 1990 to 0.85 in 2017). Our analysis showed that males were more likely to have ALL (incidence cases of ALL in male/female = 1.54:1 in 1990 and 1.41 in 2017). Subgroup analysis by SDI showed that the ASIR of ALL gradually decreased in high SDI countries during the 28 years (EAPC = -1.89, 95% CI -1.94 ~ -1.83) (Figure 1A–1C). Contrarily, the ASIR of ALL was relatively stable in middle SDI countries (EAPC = 0.82, 95% CI 0.73 ~ 0.90). Subgroup analysis by geographical zone demonstrated that Australasia had the fastest reduction in ASIR (per 100,000 individuals) (EAPC = -2.33, 95% CI -2.53 ~ -2.13). On the contrary, Andean Latin America had the most pronounced growth in ASIR (EAPC = 1.51, 95% CI 1.37 ~ 1.64).

**Table 1:** The incidence of ALL in 1990/2017 and temporal trends.

	**1990**		**2017**		**1990-2017**
	Incident cases No *10^3^ (95% UI)	**ASIR /100,000 No. (95% UI)**	Incident cases No *10^3^ (95% UI)	**ASIR /100,000 No. (95% UI)**	**EAPC No. (95% CI)**
**Overall**	49.07 (41.00~67.32)	0.89 (0.76~1.20)	64.19 (56.39~70.40)	0.85 (0.75~0.94)	-0.08 (-0.15~-0.02)
**Sex**					
**Male**	29.77 (22.72~38.73)	1.08 (0.85~1.37)	37.58 (32.24~41.52)	1.00 (0.86~1.11)	-0.23 (-0.3~-0.16)
**Female**	19.30 (14.81~32.54)	0.71 (0.55~1.16)	26.62 (21.80~30.33)	0.71 (0.58~0.82)	0.15 (0.08~0.22)
**Socio-demographic factor**					
**High SDI**	8.45 (8.27~8.69)	0.89 (0.86~0.91)	7.13 (6.92~7.43)	0.55 (0.53~0.57)	-1.89 (-1.94~-1.83)
**High-middle SDI**	11.38 (10.10~13.63)	1.03 (0.92~1.24)	12.94 (10.76~13.90)	0.97 (0.80~1.05)	-0.08 (-0.20~0.05)
**Middle SDI**	14.49 (12.34~18.94)	0.93 (0.80~1.19)	22.06 (18.45~23.71)	1.10 (0.93~1.18)	0.82 (0.73~0.90)
**Low-middle SDI**	8.30 (6.21~12.78)	0.72 (0.56~1.05)	12.70 (11.06~15.18)	0.74 (0.65~0.89)	0.23 (0.13~0.34)
**Low SDI**	6.24 (3.45~13.67)	0.74 (0.45~1.45)	9.09 (7.79~11.24)	0.65 (0.56~0.80)	-0.52 (-0.6~-0.43)
**Region**					
**Andean Latin America**	0.49 (0.39~0.67)	1.15 (0.94~1.53)	0.97 (0.73~1.13)	1.58 (1.20~1.83)	1.51 (1.37~1.64)
**Australasia**	0.33 (0.31~0.34)	1.55 (1.48~1.62)	0.32 (0.29~0.36)	0.93 (0.84~1.04)	-2.33 (-2.53~-2.13)
**Caribbean**	0.37 (0.30~0.57)	1.00 (0.82~1.50)	0.36 (0.31~0.41)	0.81 (0.69~1.06)	-0.81 (-0.92~-0.69)
**Central Asia**	0.73 (0.66~0.82)	0.97 (0.89~1.08)	0.77 (0.69~0.85)	0.88 (0.79~0.96)	-0.13 (-0.24~-0.01)
**Central Europe**	1.34 (1.26~1.44)	1.10 (1.03~1.19)	0.88 (0.83~0.93)	0.68 (0.63~0.72)	-1.69 (-1.79~-1.59)
**Central Latin America**	2.99 (2.86~3.31)	1.77 (1.71~1.92)	5.31 (5.06~5.70)	2.12 (2.02~2.27)	0.80 (0.67~0.94)
**Central Sub-Saharan Africa**	0.22 (0.09~0.56)	0.32 (0.17~0.70)	0.49 (0.31~0.86)	0.35 (0.25~0.56)	0.36 (0.31~0.42)
**East Asia**	9.06 (7.03~13.64)	0.73 (0.57~1.11)	13.68 (9.60~15.10)	0.96 (0.68~1.07)	1.29 (0.85~1.73)
**Eastern Europe**	3.26 (2.95~3.54)	1.50 (1.34~1.61)	2.32 (2.21~2.46)	1.00 (0.94~1.07)	-1.61 (-1.71~-1.50)
**Eastern Sub-Saharan Africa**	1.86 (1.06~3.43)	0.73 (0.45~1.25)	3.69 (2.76~5.15)	0.77 (0.59~1.05)	0.002 (0.15~0.31)
**High-income Asia Pacific**	1.51 (1.44~1.59)	0.91 (0.86~0.95)	1.16 (1.09~1.22)	0.50 (0.46~0.53)	-2.27 (-2.39~-2.16)
**High-income North America**	2.17 (2.12~2.22)	0.78 (0.76~0.80)	2.13 (2.02~2.25)	0.54 (0.51~0.57)	-1.47 (-1.56~-1.39)
**North Africa and Middle East**	3.28 (2.33~5.33)	0.87 (0.62~1.35)	4.28 (3.51~5.07)	0.72 (0.59~0.85)	-0.48 (-0.60~-0.36)
**Oceania**	0.04 (0.02~0.06)	0.56 (0.36~0.91)	0.07 (0.04~0.11)	0.52 (0.35~0.82)	-0.21 (-0.25~-0.16)
**South Asia**	9.76 (6.69~17.14)	0.78 (0.56~1.29)	13.01 (10.86~15.03)	0.74 (0.61~0.85)	-0.17 (-0.32~-0.02)
**Southeast Asia**	4.88 (3.65~7.61)	1.06 (0.83~1.56)	8.03 (6.84~8.93)	1.28 (1.09~1.43)	0.83 (0.63~1.03)
**Southern Latin America**	0.50 (0.46~0.54)	1.00 (0.92~1.07)	0.58 (0.53~0.64)	0.91 (0.83~1.00)	-0.23 (-0.32~-0.14)
**Southern Sub-Saharan Africa**	0.08 (0.06~0.10)	0.16 (0.12~0.18)	0.12 (0.08~0.15)	0.16 (0.11~0.19)	-0.10 (-0.47~0.27)
**Tropical Latin America**	1.73 (1.58~1.85)	1.06 (0.98~1.13)	1.85 (1.76~1.97)	0.90 (0.85~0.95)	-0.37 (-0.57~-0.17)
**Western Europe**	3.51 (3.43~3.60)	0.91 (0.89~0.94)	2.73 (2.60~2.87)	0.54 (0.51~0.57)	-2.14 (-2.28~-2.01)
**Western Sub-Saharan Africa**	0.96 (0.65~1.44)	0.45 (0.34~0.61)	1.45 (1.13~2.04)	0.35 (0.27~0.46)	-1.36 (-1.62~-1.11)

Note: ASIR: age-standardized incidence rate

**Figure 2:**
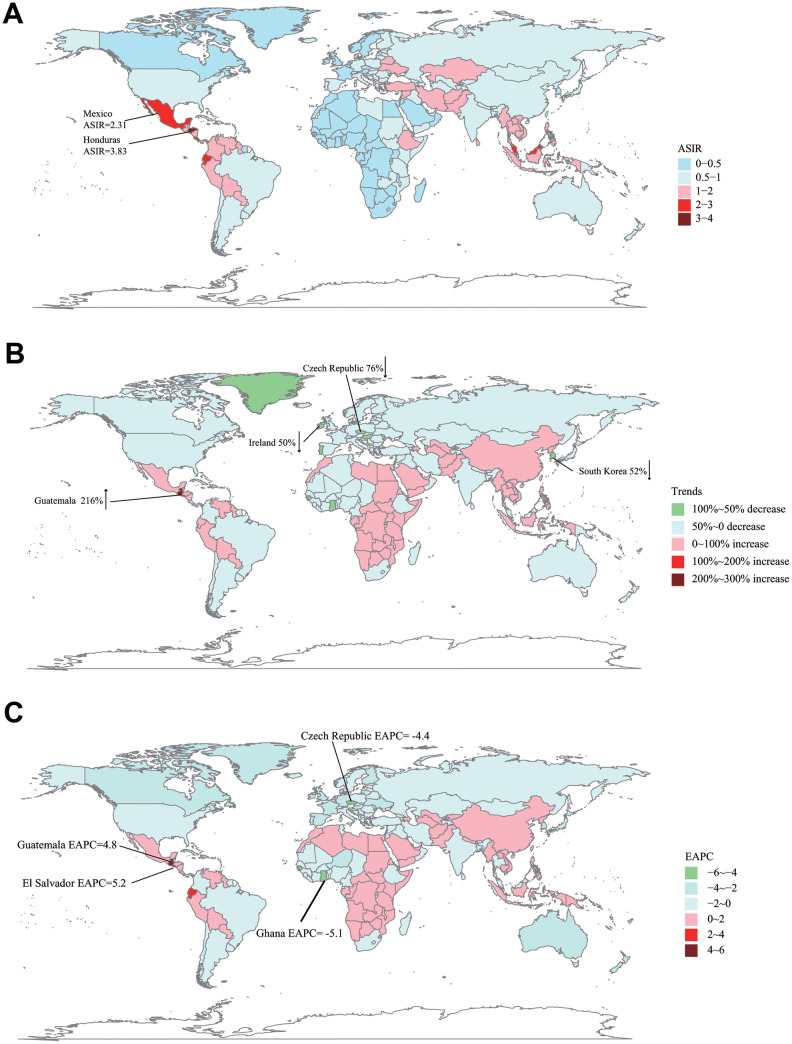
**The global disease burden of ALL in 195 countries or territories.** (**A**) The ASIR of ALL in 2017. (**B**) The relative incidences change of ALL between 1990 and 2017. (**C**) The EAPC of ALL's ASIR from 1990 to 2017. ASIR: age-standardized incidence rate; EAPC: estimated annual percentage change.

As for a specific country/territory, Namibia (ASIR = 0.12), South Africa (ASIR = 0.15), and Botswana (ASIR = 0.15) were top 3 countries with the lowest ASIR while Honduras (ASIR = 3.82), Mexico (ASIR = 2.31), and Dominica (ASIR = 2.25) were top 3 countries with the highest ASIR in 2017 (Figure 2A). Compared with ASIR in 1990, Czech Republic (75.58%), Moldova (62.13%), and Ghana (60.23%) were top 3 countries with the most percentage drop while Guatemala (216.34%), El Salvador (192.10%), and Ecuador (93.35%) were top 3 countries with the most percentage increase in ASIR in 2017 (Supplementary Tables 1, 2) (Figure 2B). Lastly, we analyzed the incidence trend in all 195 countries/territories. Ghana (EAPC = -5.18, 95% CI -6.23 ~ -4.11), Czech Republic (EAPC = -4.41, 95% CI -4.95 ~ -3.87), Guam (EAPC = -3.62, 95% CI -3.90 ~ -3.33) were top 3 countries/territories with the fastest reduction in ASIR (per 100,000 individuals). In the meanwhile, El Salvador (EAPC = 5.20, 95% CI 4.34 ~ 6.07), Guatemala (EAPC = 4.81, 95% CI 4.50 ~5.12), Ecuador (EAPC = 3.25, 95% CI 2.92 ~ 3.57) were top 3 countries/territories with the most rapid rise in ASIR (Supplementary Tables 3, 4) (Figure 2C).

### The death trends of ALL

Globally, ALL-caused death cases increased from 37.26*10^3^ in 1990 to 52.22*10^3^ in 2017 (about 40.15% rise) (Table 2). In contrast, the change trend of ASDR was stable (ASDR: from 0.70 in 1990 to 0.69 in 2017; EAPC = 0.004, 95% CI -0.06 ~ 0.07). Subgroup analysis by SDI showed that the ASDR decreased markedly in high SDI countries (ASDR: from 0.72 in 1990 to 0.46 in 2017; EAPC = -1.76, 95% CI -1.81 ~ -1.70) during past 28 years. In the same time, ASDR grew rapidly in middle SDI countries (ASDR: from 0.73 in 1990 to 0.89 in 2017, EAPC = 0.92, 95% CI 0.83 ~ 1.01) (Figure 1D–1F). In terms of geographical zone, Australasia (EAPC = -2.17, 95% CI -2.38 ~ -1.97) and high-income Asia Pacific (EAPC = -2.05, 95% CI -2.16 ~ -1.93) had the most rapid drop in ASDR while Andean Latin America (EAPC = 1.59, 95% CI 1.46 ~ 1.72) had the fastest increase in ASDR from 1990 to 2017.

**Table 2:** The death cases of ALL in 1990/2017 and temporal trends.

	**1990**	**2017**	**1990-2017**
Death cases No *10^3^ (95% UI)	**ASDR /100,000 No. (95% UI)**	Death cases No *10^3^ (95% UI)	**ASDR /100,000 No. (95% UI)**	**EAPC No. (95% CI)**
**Overall**	37.26 (31.88~49.39)	0.70 (0.61~0.91)	52.22 (45.97~56.73)	0.69 (0.61~0.75)	0.004 (-0.06~0.07)
**Sex**					
**Male**	22.49 (17.83~28.01)	0.86 (0.70~1.04)	30.60 (26.56~33.76)	0.81 (0.71~0.90)	-0.12 (-0.19~-0.05)
**Female**	14.77 (11.74~23.64)	0.56 (0.45~0.86)	21.62 (17.61~24.26)	0.57 (0.46~0.64)	0.19 (0.12~0.26)
**Socio-demographic factor**					
**High SDI**	7.21 (7.07~7.39)	0.72 (0.70~0.74)	6.68 (6.48~6.94)	0.46 (0.45~0.48)	-1.76 (-1.81~-1.70)
**High-middle SDI**	8.73 (7.79~10.30)	0.81 (0.72~0.95)	10.97 (9.13~11.76)	0.78 (0.65~0.84)	0.06 (-0.07~0.20)
**Middle SDI**	10.81 (9.35~14.01)	0.73 (0.64~0.92)	18.11 (15.05~19.47)	0.89 (0.74~0.96)	0.92 (0.83~1.01)
**Low-middle SDI**	6.01 (4.57~8.97)	0.55 (0.44~0.78)	9.64 (8.41~11.42)	0.59 (0.51~0.69)	0.35 (0.25~0.45)
**Low SDI**	4.36 (2.50~9.09)	0.55 (0.35~1.03)	6.60 (5.65~8.13)	0.50 (0.43~0.61)	-0.43 (-0.52~-0.34)
**Region**					
**Andean Latin America**	0.36 (0.29~0.48)	0.90 (0.74~1.15)	0.76 (0.57~0.87)	1.26 (0.95~1.43)	1.59 (1.46~1.72)
**Australasia**	0.29 (0.28~0.31)	1.34 (1.29~1.41)	0.32 (0.28~0.36)	0.83 (0.75~0.93)	-2.17 (-2.38~-1.97)
**Caribbean**	0.27 (0.23~0.39)	0.76 (0.65~1.06)	0.29 (0.25~0.36)	0.63 (0.55~0.80)	-0.73 (-0.84~-0.62)
**Central Asia**	0.54 (0.49~0.60)	0.75 (0.68~0.82)	0.60 (0.54~0.66)	0.70 (0.63~0.76)	0.02 (-0.10~0.14)
**Central Europe**	1.12 (1.05~1.20)	0.89 (0.83~0.95)	0.82 (0.78~0.86)	0.57 (0.53~0.60)	-1.49 (-1.60~-1.39)
**Central Latin America**	2.23 (2.15~2.38)	1.42 (1.38~1.49)	4.27 (4.09~4.52)	1.72 (1.64~1.82)	0.84 (0.72~0.95)
**Central Sub-Saharan Africa**	0.15 (0.07~0.37)	0.24 (0.15~0.50)	0.35 (0.23~0.57)	0.27 (0.20~0.41)	0.48 (0.41~0.54)
**East Asia**	6.76 (5.32~10.44)	0.56 (0.44~0.86)	11.63 (8.13~12.80)	0.77 (0.54~0.85)	1.46 (1.01~1.91)
**Eastern Europe**	2.64 (2.41~2.86)	1.17 (1.06~1.26)	2.09 (2.00~2.20)	0.84 (0.80~0.89)	-1.32 (-1.43~-1.21)
**Eastern Sub-Saharan Africa**	1.25 (0.73~2.25)	0.53 (0.34~0.89)	2.53 (1.90~3.47)	0.57 (0.44~0.75)	0.20 (0.06~0.34)
**High-income Asia Pacific**	1.26 (1.21~1.32)	0.73 (0.70~0.77)	1.14 (1.08~1.19)	0.42 (0.40~0.45)	-2.05 (-2.16~-1.93)
**High-income North America**	1.83 (1.79~1.87)	0.64 (0.62~0.65)	1.93 (1.83~2.04)	0.45 (0.43~0.48)	-1.39 (-1.46~-1.31)
**North Africa and Middle East**	2.37 (1.69~3.79)	0.67 (0.48~1.01)	3.26 (2.68~3.86)	0.57 (0.47~0.67)	-0.38 (-0.49~-0.27)
**Oceania**	0.03 (0.02~0.04)	0.43 (0.29~0.67)	0.05 (0.03~0.08)	0.40 (0.28~0.62)	-0.21 (-0.25~-0.18)
**South Asia**	7.04 (5.01~11.83)	0.59 (0.44~0.93)	9.90 (8.28~11.38)	0.57 (0.48~0.66)	-0.09 (-0.23~0.06)
**Southeast Asia**	3.69 (2.85~5.52)	0.87 (0.69~1.22)	6.57 (5.54~7.30)	1.06 (0.90~1.18)	0.89 (0.70~1.08)
**Southern Latin America**	0.39 (0.36~0.41)	0.77 (0.72~0.83)	0.49 (0.45~0.53)	0.73 (0.67~0.80)	-0.11 (-0.23~0.002)
**Southern Sub-Saharan Africa**	0.06 (0.05~0.07)	0.13 (0.10~0.15)	0.09 (0.07~0.11)	0.13 (0.09~0.16)	-0.13 (-0.50~0.24)
**Tropical Latin America**	1.25 (1.14~1.32)	0.80 (0.74~0.84)	1.48 (1.41~1.54)	0.70 (0.67~0.73)	-0.24 (-0.44~-0.05)
**Western Europe**	3.04 (2.96~3.12)	0.74 (0.72~0.76)	2.59 (2.46~2.71)	0.45 (0.43~0.48)	-1.99 (-2.13~-1.84)
**Western Sub-Saharan Africa**	0.68 (0.48~0.99)	0.36 (0.28~0.46)	1.07 (0.84~1.45)	0.29 (0.22~0.38)	-1.19 (-1.42~-0.96)

Note: ASDR: age-standardized death rate

### The DALY trends of ALL

Compared to DALY in 1990, global DALY increased by 18.56% to 2704.69*10^3^ in 2017. Age-standardized DALY (per 100,000 individuals) was slightly decreased from 39.67 in 1990 to 36.67 in 2017 (EAPC = -0.20, 95% CI -0.27 ~ -0.12) (Table 3). Subgroup analysis by SDI indicated that high SDI region possessed the fastest decline in age-standardized DALY (EAPC = -2.10, 95% CI -2.15 ~ -2.04) but middle SDI region had the most rapid increase (EAPC = 0.69, 95% CI 0.60 ~ 0.79) (Figure 1G–1I). Among 21 geographical zones all over the world, high-income Asia Pacific (EAPC = -2.61, 95% CI -2.72 ~ -2.50) and Australasia (EAPC = -2.55, 95% CI -2.75 ~ -2.35) were top 2 regions with the fastest drop in age-standardized DALY. Instead, Andean Latin America (EAPC = 1.43, 95% CI 1.29 ~ 1.56) had the most significant rise in age-standardized DALY.

**Table 3:** The DALY of ALL in 1990/2017 and temporal trends.

	**1990**		**2017**		**1990-2017**
	DALY No. *10^3^ (95% UI)	**Age-standardized DALY / 100,000 No. (95% UI)**	DALY No *10^3^ (95% UI)	**Age-standardized DALY / 100,000 No. (95% UI)**	**EAPC No. (95% CI)**
**Overall**	2281.27 (1866.34~3220.04)	39.67 (32.86~55.12)	2704.69 (2375.06~2985.23)	36.67 (32.24~40.68)	-0.20 (-0.27~-0.12)
**Sex**					
**Male**	1390.01 (1029.04~1830.71)	47.68 (36.11~61.70)	1579.13 (1355.28~1743.01)	42.17 (36.24~46.72)	-0.39 (-0.46~-0.31)
**Female**	891.27 (667.74~1560.35)	31.48 (23.91~54.15)	1125.56 (922.93~1310.01)	31.09 (25.54~36.36)	0.09 (0.01~0.17)
**Socio-demographic factor**					
**High SDI**	339.57 (331.09~349.64)	38.70 (37.63~39.94)	239.10 (230.53~249.12)	22.66 (21.71~23.64)	-2.10 (-2.15~-2.04)
**High-middle SDI**	517.96 (454.45~628.28)	46.38 (40.76~56.22)	507.74 (419.26~549.36)	42.10 (34.26~46.01)	-0.17 (-0.31~-0.03)
**Middle SDI**	687.21 (579.18~923.78)	41.02 (34.90~54.5)	916.13 (770.87~988.43)	47.12 (39.67~50.93)	0.69 (0.60~0.79)
**Low-middle SDI**	413.34 (302.70~643.1)	32.93 (24.87~49.55)	586.29 (510.05~704.70)	32.48 (28.27~38.78)	0.04 (-0.08~0.16)
**Low SDI**	314.94 (168.97~697.18)	34.27 (19.79~70.51)	445.33 (380.64~554.62)	29.45 (25.27~36.51)	-0.61 (-0.71~-0.51)
**Region**					
**Andean Latin America**	24.37 (19.06~34.23)	53.08 (42.44~72.1)	44.34 (33.00~51.95)	70.97 (52.88~82.79)	1.43 (1.29~1.56)
**Australasia**	12.01 (11.45~12.66)	60.80 (57.78~64.48)	10.12 (9.13~11.32)	34.81 (31.49~39.04)	-2.55 (-2.75~-2.35)
**Caribbean**	17.53 (14.08~27.77)	45.74 (37.12~71.14)	15.71 (13.27~20.67)	36.53 (30.54~49.02)	-0.89 (-0.99~-0.78)
**Central Asia**	35.84 (32.06~40.65)	45.46 (41.00~51.02)	35.29 (31.48~39.32)	39.36 (35.17~43.76)	-0.28 (-0.39~-0.18)
**Central Europe**	55.26 (51.23~59.83)	47.73 (44.18~51.82)	29.56 (27.72~31.30)	27.66 (25.39~29.71)	-1.93 (-2.02~-1.84)
**Central Latin America**	143.52 (137.57~156.38)	76.65 (73.83~82.41)	227.53 (216.03~242.72)	89.55 (84.97~95.53)	0.73 (0.60~0.87)
**Central Sub-Saharan Africa**	11.30 (4.57~29.05)	14.82 (7.19~34.71)	24.75 (15.69~43.46)	16.03 (10.84~26.41)	0.32 (0.25~0.38)
**East Asia**	416.76 (317.58~657.93)	32.68 (24.97~51.86)	529.14 (372.28~585.97)	42.26 (29.42~47.26)	1.18 (0.72~1.64)
**Eastern Europe**	139.99 (125.09~151.47)	68.31 (60.44~74.21)	81.17 (76.46~86.65)	40.94 (38.01~44.35)	-2.06 (-2.17~-1.95)
**Eastern Sub-Saharan Africa**	94.70 (53.56~175.08)	34.21 (20.22~60.53)	186.53 (138.07~259.91)	36.12 (27.18~49.32)	0.12 (-0.03~0.28)
**High-income Asia Pacific**	64.00 (60.80~67.70)	40.53 (38.23~43.22)	35.50 (33.26~38.10)	20.34 (18.84~22.25)	-2.61 (-2.72~-2.50)
**High-income North America**	87.52 (85.12~89.67)	34.00 (33.03~34.89)	75.07 (71.20~79.57)	22.03 (20.91~23.26)	-1.72 (-1.82~-1.62)
**North Africa and Middle East**	160.79 (113.30~269.29)	39.32 (28.00~63.19)	196.97 (162.74~236.91)	32.07 (26.44~38.44)	-0.51 (-0.65~-0.38)
**Oceania**	1.77 (1.12~3.08)	24.02 (15.47~40.04)	3.09 (2.04~5.25)	22.36 (15.02~36.42)	-0.18 (-0.23~-0.13)
**South Asia**	491.35 (332.57~868.42)	36.67 (25.91~62.36)	604.18 (502.57~700.64)	33.02 (27.47~38.31)	-0.30 (-0.48~-0.13)
**Southeast Asia**	228.38 (167.55~365.11)	44.97 (34.04~69.41)	335.7 (289.99~372.39)	52.85 (45.92~58.67)	0.73 (0.54~0.93)
**Southern Latin America**	23.49 (21.67~25.34)	46.16 (42.59~49.77)	24.74 (22.54~27.39)	40.69 (36.97~45.36)	-0.33 (-0.42~-0.24)
**Southern Sub-Saharan Africa**	3.97 (2.99~4.72)	6.77 (5.24~8.04)	5.36 (3.80~6.78)	6.76 (4.82~8.50)	-0.11 (-0.48~0.26)
**Tropical Latin America**	84.96 (76.76~91.08)	49.16 (44.70~52.51)	81.03 (76.62~85.13)	40.49 (37.99~42.71)	-0.41 (-0.63~-0.19)
**Western Europe**	137.7 (133.70~142.02)	40.17 (38.79~41.67)	90.08 (85.56~95.47)	22.31 (21.08~24.02)	-2.37 (-2.50~-2.23)
**Western Sub-Saharan Africa**	46.07 (30.87~71.93)	19.18 (13.72~27.37)	68.83 (53.83~98.20)	14.07 (11.00~19.00)	-1.57 (-1.86~-1.28)

Note: DALY, Disability-Adjusted Life Years

### The correlation between social development degree and ALL's incidence or mortality trends

**Figure 3:**
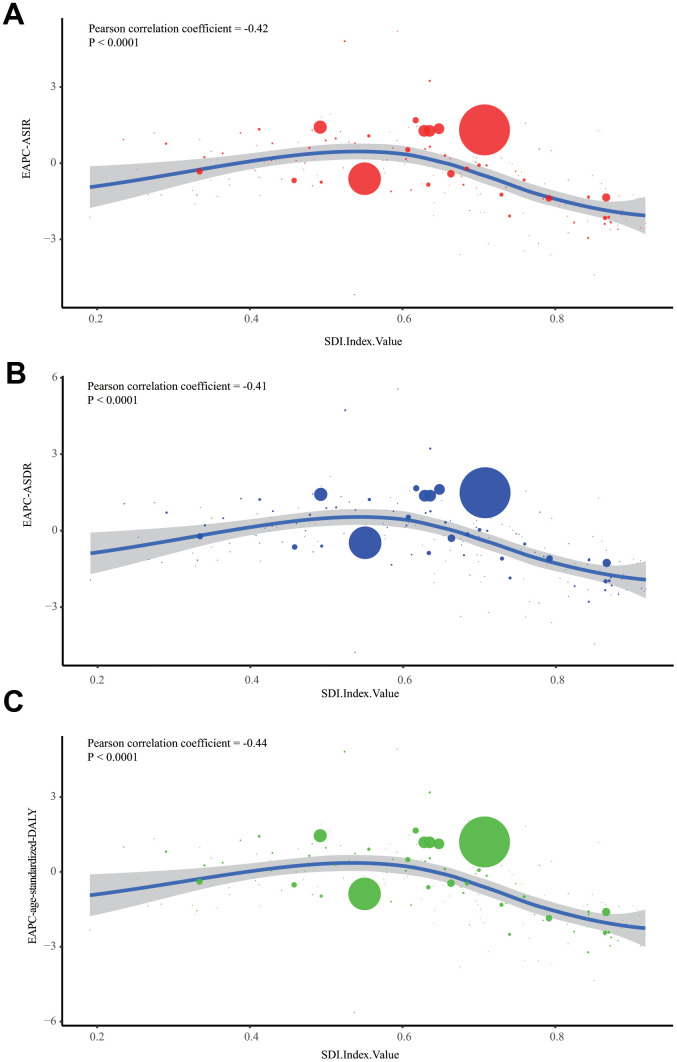
**The correlation between EAPCs and SDI in 2017.** (**A**) The correlation of ALL's EAPC of ASIR and SDI in 2017. (**B**) The correlation of ALL's EAPC of ASDR and SDI in 2017. (**C**) The correlation of ALL's EAPC of age-standardized DALY rate and SDI in 2017. One circle refers to one country, and the size of the circle reflects the quantity of ALL cases. SDI: Socio-demographic Index; ASIR: age-standardized incidence rate; ASDR: age-standardized death rate; EAPC: estimated annual percentage change;

**Figure 4:**
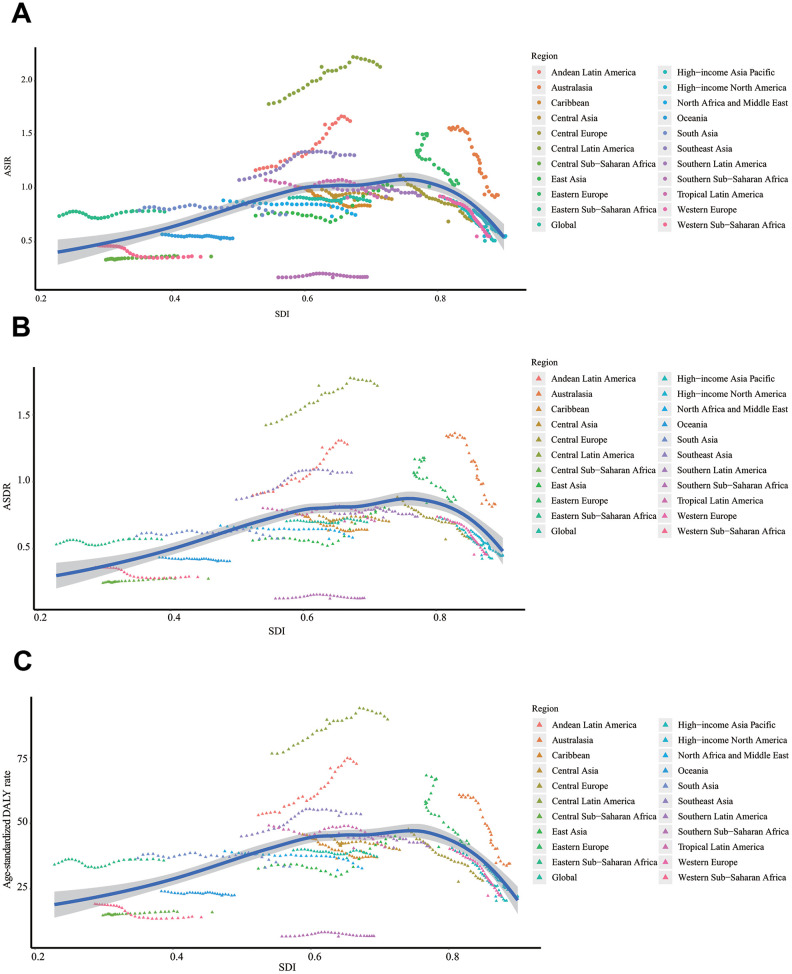
**The change trends of ASRs and SDI from 1990 to 2017 in the globe.** (**A**) The change trends of ASIR and SDI from 1990 to 2017. (**B**) The change trends of ASDR and SDI from 1990 to 2017. (**C**) The change trends of age-standardized DALY rate and SDI from 1990 to 2017. ASIR: age-standardized incidence rate; ASDR: age-standardized death rate; EAPC: estimated annual percentage change; DALY: disability-adjusted life year; SDI: Socio-demographic Index.

We constructed a Pearson's correlation model to assess the relationship between social development degree and ALL's incidence or mortality trends. Generally, the EAPC values of ASIR (Pearson correlation coefficient, abbreviated to r = -0.42, P < 0.0001), ASDR (r = -0.41, P < 0.0001), as well as age-standardized DALY (r = -0.44, P < 0.0001) were negatively correlated to SDI (Figure 3A–3C). It was notable that this decline was most significant when the SDI value was above 0.6. To further investigate the role of social development degree in ALL's incidence or mortality trends, we drew the scatter diagrams to present dynamic changes of SDI and ASRs (ASIR, ASDR, and age-standardized DALY) of 21 regions in the globe during past 28 years (Figure 4A–4C). We found that for most regions with middle or low SDI (expect Andean Latin America and Central Latin America), the ASRs were relatively stable. However, for most regions with high SDI, the ASRs were dramatically decreased, accompanied by gradually elevated SDI.

### Age distribution of ALL

**Figure 5:**
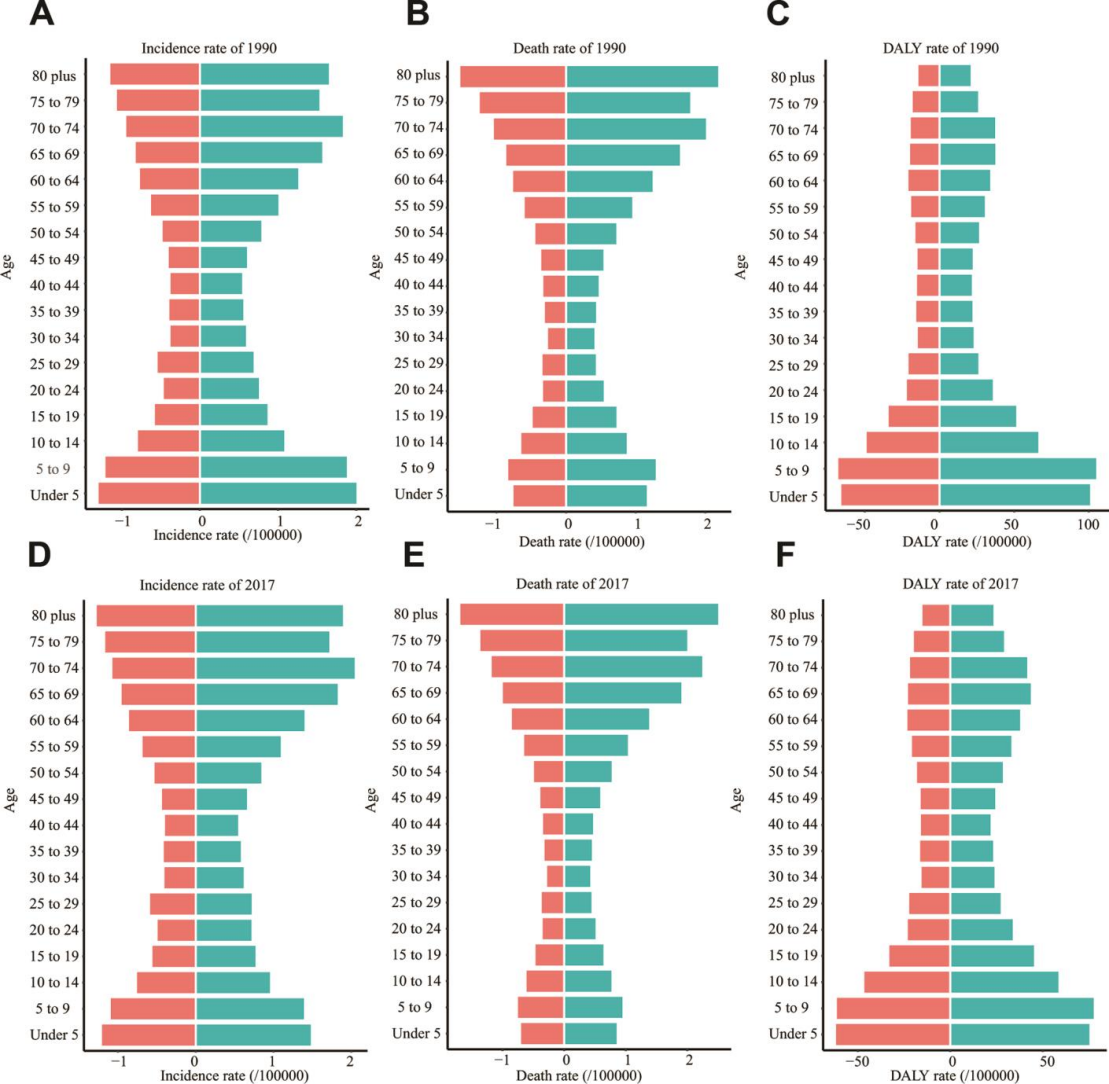
**The incidence rate, death rate, and DALY rate of ALL in different age groups.** (**A**) The incidence rate in 1990. (**B**) The death rate in 1990. (**C**) DALY rate in 1990. (**D**) The incidence rate in 2017. (**E**) The death rate in 2017. (**F**) DALY rate in 2017. EAPC: estimated annual percentage change; DALY: disability-adjusted life year; SDI: Socio-demographic Index.

Firstly, we presented the incidence rate of ALL in all age groups (Figure 5A, 5D). We found that the incidence rate of ALL had two peaks: children (under five years old) and the elderly. Almost in all age groups, males had a higher incidence rate than females. Besides, the elderly had the highest death rate (Figure 5B, 5E), while children had the highest DALY rate (Figure 5C, 5F). Compared to 1990, the incidence rate and death rate of ALL in children were reduced in 2017.

### ALL attributable risk factors

**Figure 6:**
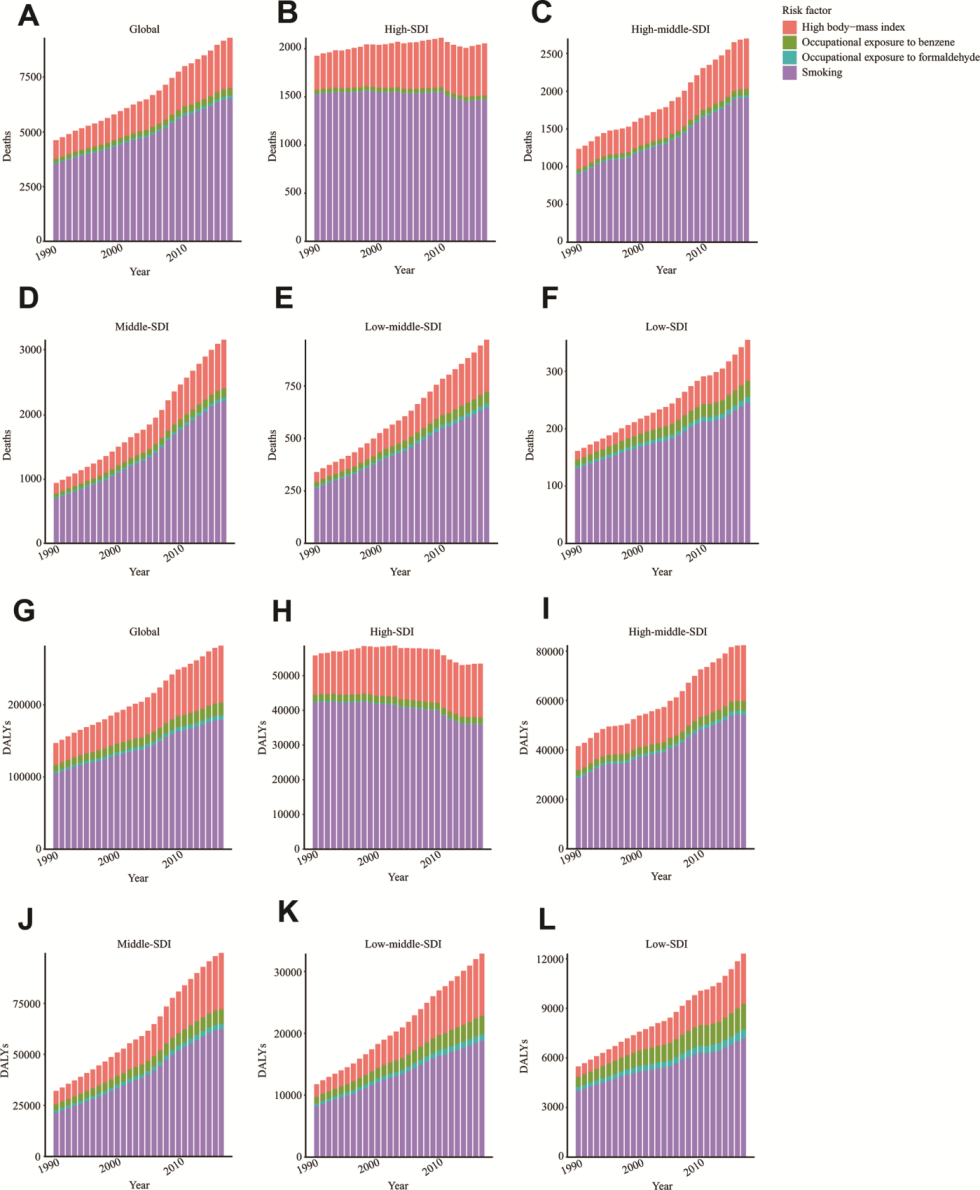
**Risk factors contributing to ALL-caused death and DALY.** Risk factors contributing to ALL-caused death in the globe (**A**), high SDI region (**B**), high-middle SDI region (**C**), middle SDI region (**D**), low-middle SDI region (**E**), and low SDI region (**F**). Risk factors contributing to ALL-caused DALY in the globe (**G**), high SDI region (**H**), high-middle SDI region (**I**), middle SDI region (**J**), low-middle SDI region (**K**), and low SDI region (**L**). DALY: disability-adjusted life year; SDI: Socio-demographic Index.

For the world and regions with different SDI values, smoking was the most significant contributor to ALL-caused death and DALY (Figure 6A–6L). However, the role of tobacco in ALL-caused death and DALY was gradually declined (Supplementary Figures 1, 2). High body-mass index was the second most significant risk factor for ALL-caused death and DALY. Notably, the contributing ratio of the high body-mass index was increased in all regions. Occupational exposure to carcinogens, including benzene and formaldehyde, was the second most significant risk factor for ALL-caused death and DALY. We found that the contributing ratio of occupational exposure to carcinogens was markedly higher in the low SDI region than the high SDI region (Supplementary Figures 1, 2).

## DISCUSSION

Our study reported the status and trend of ALL's incidence and mortality in the globe from 1990 to 2017. Considering the change of population size and distribution structure, the incidence rate and death rate of ALL were relatively stable during past 28 years. Despite this, we still noticed that the disparities of the region, gender, and age existed in ALL's incidence and mortality. Besides, the investigation of ALL's attributable risk factors revealed four factors contributing to ALL caused death and DALY. This study was helpful for the policymaker to rationally allocate public health resources and reduce ALL-caused loss, especially for regions with mushrooming incidence rates such as Andean Latin America.

According to previous statistical data, the peak of ALL's incidence rate occurred at 3 ~ 5 years of age in the United States [16]. We found that ALL's incidence rate indeed had a single peak (under five years old) in 1990 in the world. However, due to the decreased incidence rate in children, ALL's incidence rate had two peaks in 2017: one peak in children and the other peak in the elderly. Besides, we observed that the death rate of children (under five years old) was significantly decreased from 0.96 per 100,000 individuals (1990) to 0.78 per 100,000 individuals (2017). Correspondingly, ALL's incidence rate in the elderly (above 70 years old) was slightly increased (from 1.54 to 1.77 per 100,000 individuals in 2017). Benefiting from the development of modern pediatric regimens, the ten-year survival rate of children was elevated from 11.1% (1962 ~ 1966) to 91.1% (2000 ~ 2007) [17]. At the same time, the clinical outcome of the elderly was less encouraging: for ALL patients over 55 years of age, the tolerability of intensive pediatric-derived therapy was weak, and the compliance to planned chemotherapy was relatively low [18]. The data from the SEER database showed the overall 5-year survival rate of ALL (60 ~ 69 years old) was still below 20% in the United States [19].

The ASDR and age-standardized DALY rates of ALL were gradually declined in the high SDI region but kept stable in other areas. This decline was partially attributed to the improvement in the management of ALL during the past decades. In some countries, especially developed countries, the 5-year survival rate of childhood ALL was considerably high. Nevertheless, for some low-income or middle-income countries, basic interventions for ALL were not consistently available, and the 5-year survival rate of ALL was relatively low [20]. Besides, we noticed the ASIR of ALL in high SDI region was also gradually decreased. We supposed that the decline of mortality rate in high SDI region might relate to reduced predisposing factors of ALL such as pesticide exposure and ionizing radiation.

Moreover, the ASIR, ASDR, and age-standardized DALY rates in low or middle-low regions stayed at a low level. This situation is related to several factors. A truly low incidence rate, limited cancer registration, or restricted diagnosis level could contribute to a similar statistical result. The under-diagnosis and under-registration for cancer patients might be interferences when interpreting ALL's burden in some countries [21].

We found that smoking, high body-mass index, occupational exposure to benzene, and formaldehyde were risk factors contributing to ALL-caused death and DALY. Until 2017, tobacco had been the most critical risk factor of ALL's mortality. According to the data from UK Biobank, smoking was closely related to myeloid clonal hematopoiesis and *ASXL1* mutation. It has been reported that prenatal and early-life tobacco exposure could lead to the generation of ALL-related genomic deletions [22]. Additionally, the second-hand tobacco exposure of children was related to the increased risk of ALL with RAS mutation [23]. Apart from smoking, we observed that high body-mass index was the second most significant risk factor contributing to ALL's mortality. A previously multicenter cohort study showed that overweight or obesity at diagnosis was a marker predicting early mortality for ALL patients [24]. High body-mass index was closely related to increased risk of traumatic lumbar punctures and radiographic osteonecrosis during diagnosis and treatment, which might lead to the poor outcomes of ALL patients [25, 26]. The high body-mass index affected ALL patients' survival by upregulating insulin-like growth factor-1 signaling, increasing the levels of leptin, circulating glucose, certain amino acids, free fatty acids, and promoting chronic inflammation [27]. Notably, the survivors of ALL are at high risk of obesity and obesity-related metabolic disorders such as type 2 diabetes [15, 28].

Furthermore, in this study, occupational exposure to carcinogens such as benzene and formaldehyde, also contributed to ALL-caused death or DALY. A retrospective study showed that benzene exposure during childhood was associated with increased risk of ALL and acute myeloid leukemia [29]. Even parental or maternal occupational exposure to benzene could elevate the risk of ALL in their offspring [30–32]. Moreover, it was found that formaldehyde exposure could disrupt hematopoietic function and induce leukemia-related chromosome alterations [33]. Notably, the contribution ratio of occupational exposure to carcinogens was significantly higher in low and low-middle SDI regions than the high SDI region. There was much room for improvement in reducing exposure to carcinogens for low or low-middle SDI regions.

## CONCLUSIONS

Generally, the cases of ALL were continuously increased in the past 28 years in the globe. Excluding the change in population size and structure, the global incidence rate was relatively stable. Notably, the ASRs of ALL in the high SDI region were gradually decreased from 1990 to 2017, which might be attributed to the development of ALL treatment strategy and the declined exposure to predisposing factors. For most middle/low SDI regions except Andean Latin America and Central Latin America, the ASRs of ALL kept stable. Smoking, high body-mass index, and occupational exposure to benzene and formaldehyde were the main risk factors contributing to ALL-caused mortality. Measures should be taken to reduce ALL-caused loss, including reducing exposure to tobacco and carcinogens, especially for low or middle SDI countries considering the relatively high contribution ratio of carcinogen exposure in these countries.

## MATERIALS AND METHODS

### Data acquisition

Statistical data, including ALL's incidence rate, death rate, and DALY rate, were downloaded by the Global Health Data Exchange tool (http://ghdx.healthdata.org/gbd-results-tool). Moreover, we collected some disease-associated parameters, such as SDI and age or sex distribution data. Besides, data on ALL-caused death and DALY attributable risk factors such as occupational exposure to carcinogens, smoking, and high body-mass index were extracted.

### Statistical analysis

The burden of ALL was estimated by the number of incidence cases, death cases, and DALYs from 1990 to 2017. To offset the changes in total population quantity and age distribution, the age-standardized incidence and death rate (ASIR and ASDR), as well as age-standardized DALY rate, were also used in this study. The change trends of incidence rate, death rate, DALY rate were calculated by estimated annual percentage change (EAPC) values. In the present study, EAPC was calculated based on three age-standardized rates (incidence, death, and DALY) and regression model. The following algorithm was adopted to get EAPC: *y = α + βx*, in which y referred to log10 (ASR) and x meant year. Then, EAPC was calculated as EAPC = 100*(10^β-1). The values of EAPC reflect the changing trend of corresponding ASRs: when EAPC and its 95% CI are above 0, it indicates an increasing trend; when EAPC and its 95% CI are below 0, it shows a degenerating trend. Besides, to assess the influence of social development on ALL's incidence and mortality, we plotted a scatter diagram and trend lines to present the correlation between EAPC and SDI. Lastly, GBD 2017 provides data on cancer death and DALY attributable risk factors, including environmental or occupational factors, behavior factors, and metabolic factors. The three types of factors which could be further subdivided to 84 risk factors. In this study, we found four risk factors contributing to ALL's mortality: occupational exposure to benzene, occupational exposure to formaldehyde, smoking, and high body-mass index.

### Data visualization

All calculations were based on R software (version 3.6.0). We drew world maps to reflect the incidence status and change trend of ALL in the globe. All data visualization works were conducted by R software with packages ‘ggplot2’ and ‘map’.

## Supplementary Material

Supplementary Figures

Supplementary Tables
