# Supplementary material for: Global burden and trend of acute lymphoblastic leukemia from 1990 to 2017
Source: Aging (Albany NY). 2020 Nov 16;12(22):22869–91. doi: 10.18632/aging.103982 (PMC7746341; doi:10.18632/aging.103982)
Supplement: Supplementary Figures [file aging-12-103982-s001..pdf]

SUPPLEMENTARY FIGURES

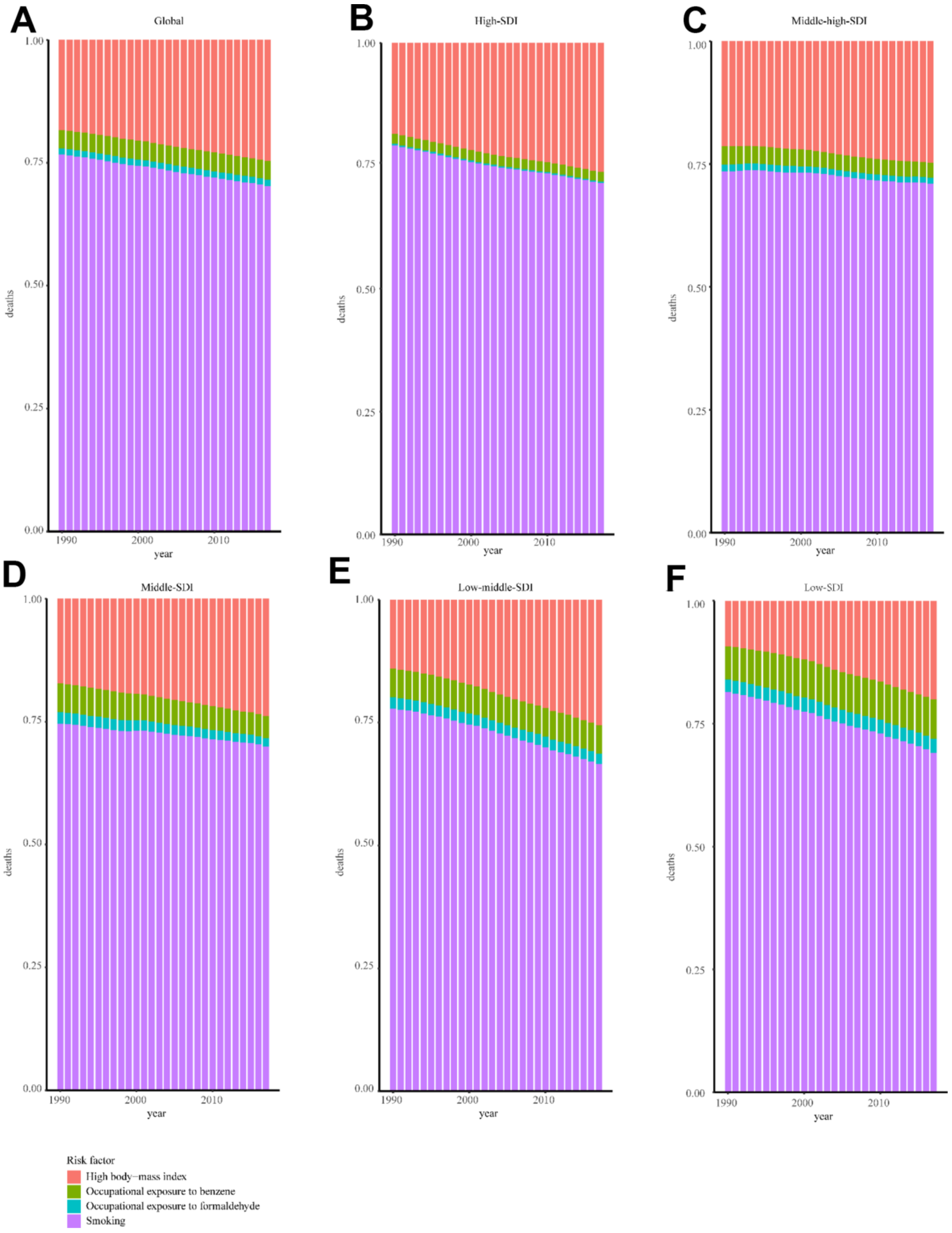

Supplementary Figure 1. The ratio of risk factors contributing to ALL-caused death in the global (**A**), high SDI region (**B**), high-middle SDI region (**C**), middle SDI region (**D**), low-middle SDI region (**E**), and low SDI region (**F**). SDI: Socio-demographic Index.

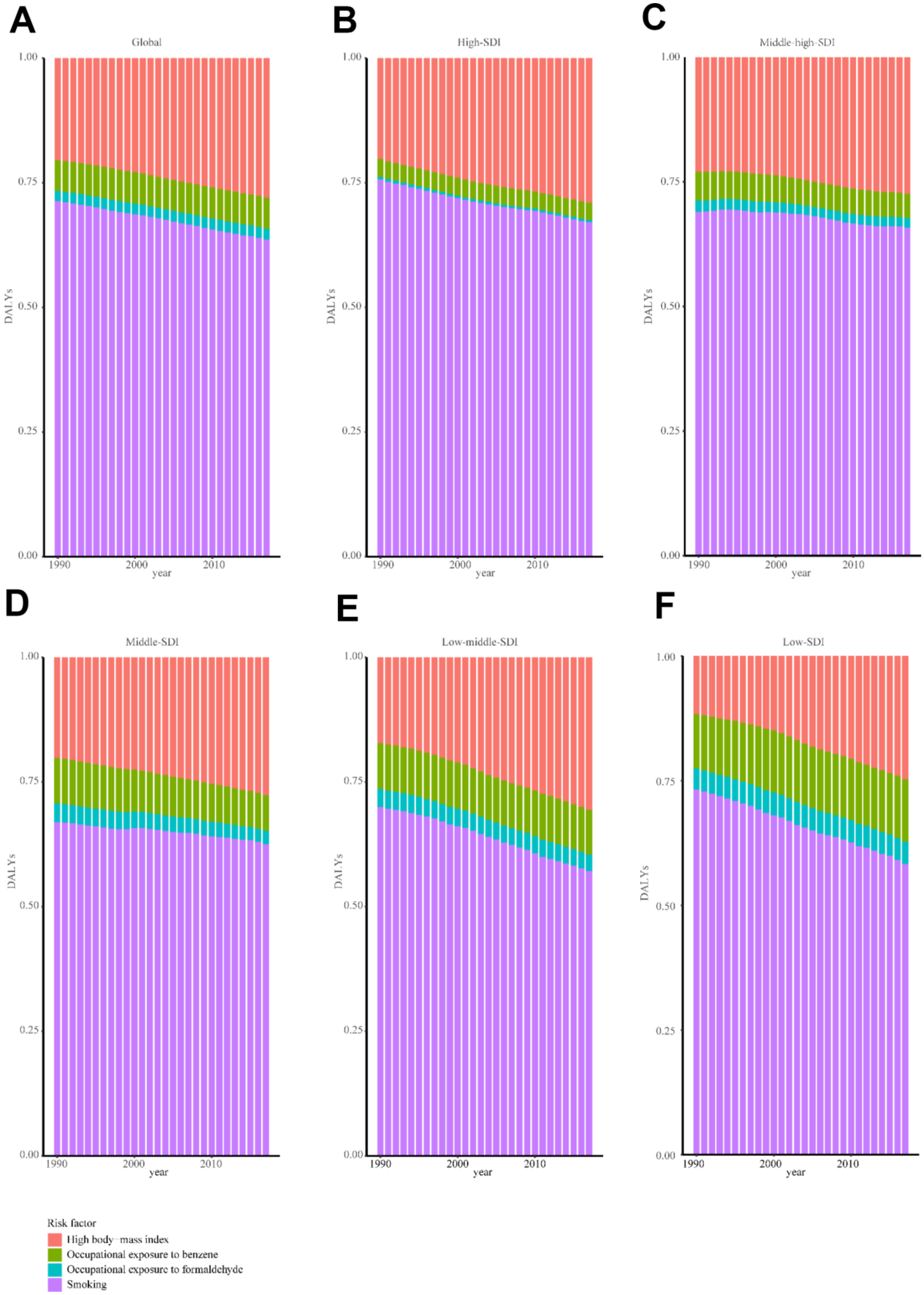

Suppelementary Figure 2. The ratio of risk factors contributing to ALL-caused DALY in the global (**A**), high SDI region (**B**), high-middle SDI region (**C**), middle SDI region (**D**), low-middle SDI region (**E**), and low SDI region (**F**). DALY: disability adjusted life year; SDI: Socio-demographic Index.
