# Supplementary material for: Global burden and trend of acute lymphoblastic leukemia from 1990 to 2017
Source: Aging (Albany NY). 2020 Nov 16;12(22):22869–91. doi: 10.18632/aging.103982 (PMC7746341; doi:10.18632/aging.103982)
Supplement: Supplementary Tables [file aging-12-103982-s002..pdf]

SUPPLEMENTARY TABLES

Supplementary Table 1. Top 30 countries/territories with the biggest drop in ASIR in 2017 compared to 1990.

Country/Territory	ASIR-1990	ASIR-2017	Change(%)
Czech Republic	1.70444118	0.416184335	-75.58235863
Moldova	2.07416161	0.78541563	-62.13334454
Ghana	0.990678333	0.393955263	-60.23378631
Greenland	1.039359543	0.4299638	-58.63185138
Guam	0.683243245	0.304279976	-55.46535179
Hungary	1.158125344	0.553921424	-52.17085722
South Korea	0.830602251	0.398224777	-52.05589964
Singapore	0.972292344	0.473770426	-51.27284208
Ireland	0.958707392	0.476495364	-50.29814438
Portugal	1.094743284	0.54648071	-50.08138271
UK	0.975371906	0.497623851	-48.98111709
Grenada	0.827295887	0.425663223	-48.54764422
Netherlands	0.858379537	0.451789964	-47.36710922
Canada	0.872223561	0.462677459	-46.95425806
Croatia	1.053279621	0.562950499	-46.55260692
France	0.860469991	0.467062191	-45.72010696
Denmark	1.16850441	0.640590085	-45.17863348
Qatar	1.189622774	0.660784651	-44.45427025
Belgium	0.765127831	0.425527438	-44.38479163
Lithuania	1.252188712	0.70092604	-44.02392917
Bahrain	0.982153076	0.550692851	-43.93003858
Luxembourg	0.723161985	0.414477003	-42.68545479
Germany	0.930893753	0.542561113	-41.7161076
Finland	1.232210269	0.718940517	-41.65439657
Bermuda	0.74033626	0.43311429	-41.49762564
Japan	0.904300875	0.53010933	-41.379098
Spain	0.920329377	0.540555299	-41.2650174
Australia	1.562040153	0.932742146	-40.286929
Israel	0.92440745	0.562882957	-39.10878181
New Zealand	1.464291458	0.904585214	-38.22369114

Supplementary Table 2. Top 30 countries/territories with the biggest rise in ASIR in 2017 compared to 1990.

Country/Territory	ASIR-1990	ASIR-2017	Change(%)
Guatemala	0.623604531	1.972724414	216.3422193
El Salvador	0.456314727	1.332917333	192.1048245
Ecuador	1.149040472	2.221646264	93.34795573
Dominica	1.403302844	2.247426347	60.15262541
Philippines	0.886534379	1.41587231	59.70867508
Timor-Leste	0.808771264	1.247081505	54.19458632
Saint Vincent	0.420853917	0.642293089	52.61663573
Grenadines	0.420853917	0.642293089	52.61663573
Lesotho	0.123348818	0.180789334	46.56754507
Pakistan	0.959267942	1.344962632	40.20719065
South Sudan	0.440881341	0.616906572	39.92576116
Somalia	0.564531942	0.787166259	39.4369744
Tanzania	0.542464015	0.754500192	39.08760246
Eritrea	0.665596439	0.915702395	37.57621608
Libya	0.547060231	0.751913167	37.44613927
Mexico	1.706860901	2.310334065	35.35573189
Cambodia	0.772675369	1.035146681	33.96915721
Indonesia	0.814414691	1.086220349	33.374356
China	0.729840663	0.97081053	33.01677747
Azerbaijan	0.654738789	0.855316688	30.63479694
Laos	0.955927443	1.248609961	30.61764997
Central African Republic	0.318516553	0.411046704	29.05034279
Uganda	0.32054807	0.413000208	28.84189518
Myanmar	1.050643081	1.333863227	26.95683721
Honduras	3.023909046	3.82808714	26.59399082
Gabon	0.307167136	0.382356481	24.47831689
Paraguay	0.846577595	1.049210003	23.93547972
Guyana	0.904207845	1.120459652	23.91616135
Kenya	0.461398528	0.570032709	23.54454437
Mauritius	0.333233622	0.410143492	23.07986492

Supplementary Table 3. Top 30 countries/territories with the most rapid rise in ASIR.

Country/territory	confidence interval (Low)	EAPC of ASIR	confidence interval (high)
El Salvador	4.34	5.201	6.069
Guatemala	4.499	4.806	5.115
Ecuador	2.923	3.245	3.569
Dominica	1.862	2.026	2.19
Timor-Leste	1.811	1.938	2.065
Lesotho	1.24	1.793	2.349
Philippines	1.338	1.694	2.051
Mauritius	0.796	1.604	2.418
Libya	1.296	1.576	1.857
Paraguay	0.99	1.444	1.9
Pakistan	1.255	1.421	1.587
Grenadines	0.824	1.404	1.987
Saint Vincent	0.824	1.404	1.987
Indonesia	1.11	1.36	1.61
Tanzania	1.187	1.337	1.486
China	0.857	1.312	1.769
Cambodia	1.152	1.298	1.445
Mexico	0.989	1.275	1.561
Brunei	1.013	1.26	1.507
Laos	1.038	1.25	1.462
Guyana	0.986	1.227	1.468
Uganda	0.931	1.196	1.461
South Sudan	0.827	1.194	1.561
Gabon	0.94	1.17	1.402
Myanmar	0.792	1.076	1.361
Azerbaijan	0.567	1.002	1.438
Honduras	0.739	0.975	1.212
Central African Republic	0.87	0.937	1.004
Somalia	0.606	0.936	1.266
Kenya	0.776	0.897	1.017

Supplementary Table 4. Top 30 countries/territories with the fastest reduction in ASIR.

Country/territory	confidence interval (Low)	EAPC of ASIR	confidence interval (high)
Ghana	-6.234	-5.175	-4.105
Czech Republic	-4.948	-4.412	-3.874
Guam	-3.904	-3.618	-3.331
Moldova	-3.796	-3.603	-3.409
Greenland	-3.658	-3.455	-3.252
Bahrain	-3.645	-3.162	-2.676
Portugal	-3.534	-3.162	-2.787
UK	-3.13	-2.946	-2.763
Qatar	-3.287	-2.857	-2.424
Hungary	-2.908	-2.742	-2.575
Singapore	-2.903	-2.614	-2.324
Canada	-2.811	-2.605	-2.399
Luxembourg	-2.799	-2.593	-2.387
Netherlands	-2.678	-2.544	-2.411
Ireland	-2.709	-2.445	-2.18
France	-2.542	-2.393	-2.244
Denmark	-2.717	-2.375	-2.031
Spain	-2.533	-2.347	-2.16
Australia	-2.557	-2.339	-2.122
South Korea	-2.954	-2.313	-1.668
Croatia	-2.505	-2.295	-2.085
New Zealand	-2.478	-2.28	-2.081
Finland	-2.565	-2.232	-1.897
Japan	-2.288	-2.157	-2.026
Germany	-2.436	-2.129	-1.821
Niger	-2.352	-2.125	-1.898
Israel	-2.465	-2.087	-1.707
Ukraine	-2.705	-2.082	-1.455
Grenada	-2.507	-2.063	-1.616
Belgium	-2.278	-2.042	-1.805
